# Indigenous knowledge for disaster risk reduction: An African perspective

**DOI:** 10.4102/jamba.v8i1.272

**Published:** 2016-07-29

**Authors:** Nnamdi G. Iloka

**Affiliations:** 1 Faculty of Engineering and Environment, Northumbria University, Newcastle City Campus, United Kingdom

## Abstract

Indigenous knowledge is valuable knowledge that has helped local communities all over the world survive for generations. This knowledge originates from the interaction between members of the community and the environment in which they live. Although much has been written about indigenous knowledge, its documentation in the area of disaster risk reduction and climate change in Africa has been very limited. The wealth of this knowledge has not been well-recognised in the disaster risk reduction field, as policy-makers still rely on mitigation strategies based on scientific knowledge. Colonialism and lack of proper documentation of indigenous knowledge are some of the contributing factors to this. Ignoring the importance of understanding adaptive strategies of the local people has led to failed projects. Understanding how local people in Africa have managed to survive and adapt for generations, before the arrival of Western education, may be the key to developing sustainable policies to mitigate future challenges. Literature used in this article, obtained from the books, papers and publications of various experts in the fields of disaster risk reduction, climate change, indigenous knowledge and adaptation, highlight the need for more interest to be shown in indigenous knowledge, especially in the developing country context. This would lead to better strategies which originate from the community level but would aim for overall sustainable development in Africa.

## Indigenous knowledge: Relevant knowledge?

Indigenous knowledge has been receiving a lot of attention since the early 1990s in the field of disaster management and in issues associated with climate change (Hiwasaki *et al*. 2014). Disaster risk reduction policies of developing countries had been known to be carried out, using scientific knowledge, while ignoring the value of indigenous knowledge (Gaillard & Mercer 2012; Rist & Dahdouh-Guebas 2006). Indigenous knowledge is usually underappreciated as it is viewed in some quarters as inferior to scientific knowledge (Mercer *et al*. 2010). However, there have been calls for increased utilisation of knowledge of the local people – knowledge which has been used for generations to mitigate hazards and reduce disasters in local communities (Gaillard & Mercer 2012; UNISDR 2005).

The importance of indigenous knowledge in the field of disaster management has continued to grow (Mercer *et al*. 2010). Its potential for improving disaster risk reduction policies has been discussed on various platforms, such as the management of disasters in the Asia-Pacific region, with reference to the Hyogo Framework for Action – Priority 3 (Shaw, Noralene & Baumwoll 2008a). Literature on the value of indigenous knowledge (Hilhorst *et al*. 2015) in disaster risk reduction highlights important points. One point, for instance, is the role indigenous knowledge plays in empowering local community members to take front roles in activities aimed at disaster risk reduction such as mixed cropping which sustains soil and leads to yield of various crops – this ensures alternative crops are available for consumption if some other crops fail (Mwaura 2008). Such activity makes local disaster management champions in their own right. It has also been argued that ways through which this knowledge is spread in communities could act as a model in education on issues of disaster risk reduction (Shaw *et al*. 2008a). In Africa, indigenous knowledge has been used in traditional medicine, agriculture and food production, engineering and ecological management for natural resources (Domfeh 2007). Indigenous knowledge has made local communities live in harmony with their environment for long periods of time – this has improved knowledge about their environment, knowledge which is acquired through the practice of coping mechanisms, methods of conservation, studying the weather and seasons prediction (Mwaura 2008). Local communities in African countries are faced with various types of hazards and to tackle such hazards, the local communities use local, community-based strategies obtained from their indigenous knowledge (Nyong, Adesina & Osman Elasha 2007).

## Indigenous knowledge

Indigenous knowledge has been passed down generations, gained from knowledge of the environment which is revealed through intuitions, dreams or visions (Agrawal 1995). In various contexts, it may be presented as local knowledge or traditional knowledge, all coming to mean the same thing (Kelman, Mercer & Gaillard 2012). It has been argued that indigenous knowledge is very important in planning for community development (Mutasa 2015) and that developmental strategies cannot be completely successful without the implementation of local knowledge (Nyong *et al*. 2007). Indigenous knowledge provides communities with ideas for tackling local problems and helps in their developmental processes. The knowledge sustains the local communities while strengthening their cultural identity (Jabulani 2007). Indigenous knowledge forms the basis for local communities coping strategies that have helped them survive over a long period of time. This knowledge has been harnessed from the interaction of indigenous people with their natural environment. It provides valuable information with regards to the local environment and can be adapted for use in tackling hazards in other local communities. Local empowerment is attained through the use of indigenous knowledge in local communities, empowerment which leads to improved community participation and educating individuals on disaster risk reduction (Shaw *et al*. 2008b). Indigenous knowledge is unique to a particular community and is stable for such a community as it has been used over a long period of time – sometimes evolving through generations (Dekens 2007).

### Indigenous knowledge in agriculture

Local cultivation of crops and rearing of livestock in Africa are largely dependent on indigenous knowledge of local people. This indigenous knowledge enhances food security through methods that pose low risks to the environment. For instance, before the popularity of industrial insecticides, plant derivatives were used by local people for insect control in crop production (Domfeh 2007). Plant derivatives are biodegradable and many cause no harm to mammals. Some of these derivatives such as oil and ash from eucalyptus and neem trees are effective against beetles (Rahman & Talukder 2006). Plants derivatives used as insecticides have also been found to contain chemicals which prevent insects from developing resistance to them (Domfeh 2007). There is a lower risk of famine that may be caused by insect infestations. Indigenous knowledge literature has shown that local crops are of medicinal value and have kept local communities healthy for generations (Shackleton & Campbell 2000). To keep livestock healthy for milk and milk production, the Maasai in Kenya use a special method in testing for immunity in their local herds during rinderpest outbreaks affecting animals in neighbouring villages. They collect blood from infected animals and smear it on the nostrils of their own herd. Animals that survive after this is done develop an immunity to the disease and cannot be re-infected (Mwaura 2008). Such local remedies highlight the importance of indigenous knowledge to agriculture and livestock production.

### Indigenous knowledge in medicine

Melchias (2001) wrote that indigenous medicinal plants account for drugs used in healthcare for over 80% of the world’s population. Medicinal plant species used in the Central African region have been found to be as efficient as Western drugs; this helps with the control of disease outbreaks and the World Health Organization recognises this immense contribution of indigenous knowledge towards world health. Western drugs manufacturing companies even invest billions of dollars in botanical gardens, like the ones located in Cameroon and the Democratic Republic of Congo, to research indigenous plants and harness knowledge (Eyong 2007; Nkuinkeu 1999).

### Indigenous knowledge in land and soil management

To manage the lands on which these important crops are planted, indigenous practices such as mixed cropping which preserves the fertility of soil to ensure availability of food, and minimal tillage which keeps top soil strong enough so as not to be washed away by flood water, are used by the local African people (Domfeh 2007; Mwaura 2008). Mixed cropping systems allows for the planting of various types of crops in a particular portion of land – for example, planting maize with beans. Such symbiotic relationship increases the fertility of soil through nitrogen fixation and helps control weeds. It is also important to note that such an indigenous practice reduces the likelihood of the occurrence of famine that may result from the failure of one particular kind of crop due to diseases or other hazards – there would be a replacement crop available for consumption (Mwaura 2008).

### Indigenous knowledge in natural resource management

Conservation of natural resources to ensure sustainability is another area where indigenous knowledge is invaluable. In West Africa, traditional forest management techniques help with forest conservation. The conservation of indigenous animal species is of paramount importance to every community. Local hunters in Africa use indigenous knowledge to preserve species indigenous to their communities by avoiding pregnant and young animals when hunting for animals that are considered a delicacy in their different communities – this ensures continuity for such species (Eyong 2007). Forests that are recognised as ‘sacred’ by the local communities are home to indigenous plant and animal species. As there is no disturbance from humans who revere such forests, these species are preserved. Such forests may be home to special plant and animal species that need continued biodiversity to survive. The forests are termed ‘sacred’ by the local communities because of religious or ancestral beliefs. The plants and animal species that make up such forests are indirectly protected from exploration, thereby giving them a lower chance of extinction. Local shrines are also a safe haven for indigenous flora and fauna (Eyong, Mufuaya & Foy 2004). In Sudan, indigenous knowledge has been used for water conservation. Wells are dug using traditional techniques and water obtained from these wells is used by local families and also used for farming. These wells can reach a depth of 50 feet in some instances and are made using local tools. The wells go a long way in ensuring adequate and needed water supply (El Sammani & Dabloub 1996:30).

### Indigenous knowledge in disaster risk management

Local people in Nigeria have used indigenous plants to tackle bank and gully erosions. Abam (1993) carried out a study in the Niger Delta region of Nigeria, which led to the knowledge that river banks, where bamboo plants and raffia palms had been planted by local people, prevented the washing away of soil which causes bank erosions. These plants serve as current breakers for the water and over time, strengthen the materials that make up the river banks. Apart from their effectiveness against gully erosion, bamboo plants are also very valuable for controlling soil erosion and preventing landslides, due to their fibrous roots and rhizome system which hold soil together (Zhou *et al*. 2005). In Swaziland, the presence of specific birds’ species on trees can indicate the onset of the rainy season for the local people and floods can be predicted by how high birds build their nests from river surfaces (Domfeh 2007).

## ‘Hard’ or ‘soft’ technology for indigenous knowledge?

Indigenous knowledge provides a wealthy store of knowledge which can be applied to initiatives from small rural communities to national platforms. It is a vast system of knowledge that includes local technical knowledge, traditional environmental knowledge and ‘science of the local people’. This knowledge has been shown to evolve with time and can grow or diminish, depending on the situation of a particular local community. Indigenous knowledge empowers local communities for their development and this empowerment is based on the application of appropriate technology in various contexts (Tharakan 2015). The debate on what constitutes appropriate technology has been around for a long time (Rybcynzski 1991) but generally it could range from minimum capital to labour intensive initiatives, to large government-sponsored projects for local communities (Tharakan 2015). The creativity of the human mind has enabled local communities to think about how to sustainably manage their environment and at the same time, ensure development. However, well-funded scientific development ideologies with goals usually profit-based, have threatened to eliminate this grassroots knowledge (Goonatilake 1984). Appropriate technology has continued to enable local communities to provide themselves with the basic necessities of life. The technology can range from the use of trees in local communities for health purposes and the aforementioned Maasai method of checking for animals’ immunity (Mwaura 2008), to more advanced procedures such as acupuncture (Tharakan 2015).

**FIGURE 1:**
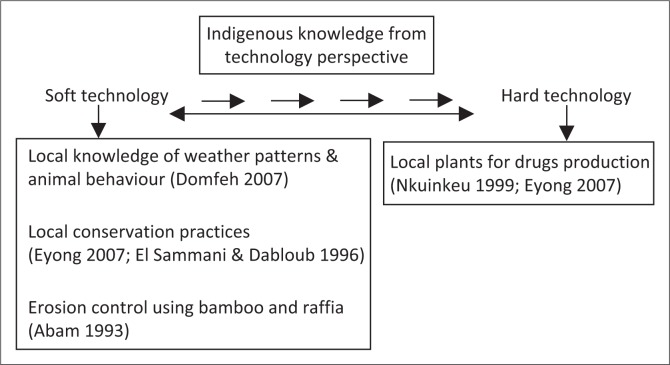
Examples of indigenous knowledge from a technology perspective.

For the purpose of this article, appropriate technology can be categorised into soft and hard technology in an indigenous knowledge context. The application of local methods to solve local problems in the community can be called soft technology. Soft technologies, which in this sense are community-led disaster risk reduction (DRR) approaches, are critical to disaster management at the local level (Abam 1993; Domfeh 2007). It involves the use of local laws and experiences to alter nature to promote human development and management of the society. It may not be presentable in a physical form, but would still be very influential for development. Hard technology on the other hand, is the application of knowledge derived from natural science that can be skilled into managing issues related to human development (Zhouying 2005). In other words, hard technology takes ideas from soft technology and advances it into more physical forms (Bessant & Francis 2005; Zhouying 2005). Bearing in mind the various types of indigenous knowledge practices that have been written about here, from the use of plant derivatives for insects’ control (Mwaura 2008) to botanical gardens where indigenous plants are studied for use in Western medicine (Eyong 2007; Nkuinkeu 1999), a scale of the relevance of indigenous knowledge as a source of appropriate technology for Africa’s development can be drawn. This scale highlights the range of technology, from the most basic practices to the more sophisticated ones, obtainable from indigenous knowledge (Figure 1).

Community-led DRR approaches save lives. When the tsunami hit the coastlines of Asian countries in 2004, indigenous communities survived through their knowledge of the environment while tourists, who did not have such local knowledge, lost their lives. The Moken community living by the coast predicted the onset of the disaster through the unusual patterns in the weather – this knowledge they had gathered through stories in the local communities that had been passed down generations (Arunotai 2008:75). The knowledge has ensured the adaptation of such coastal communities to an ever-present hazard. There is a need to advocate for adaptation strategies, rather than mitigation strategies in DRR. Local communities would continue to live in hazard-prone areas – like the Moken who live close to the ocean, the Javanese communities who live on the slopes of a volcano, due to their cultural beliefs and life patterns (Kulatunga 2011) and the people of Manjiya County in Uganda who live next to Mount Elgon, which is a basaltic volcano (Knapel *et al*. 2006). A changing climate would always pose risks to such communities and make them constantly vulnerable (Kulatunga 2011). Such communities need to stay safe in the face of constant, ever-present hazards.

## Mitigation and adaptation strategies for disaster risk reduction

There is a link between adaptation due to a changing environment and disaster risk reduction, as many hazards that lead to disasters are products of the changing climate. Changes in weather patterns and environmental factors have led to increased vulnerability of communities to hazards (Birkmann & Von Teichman 2010). The impacts are mostly felt in developing countries in Africa and Asia, as most of these countries lack the capacity to cope (Shaw, Pulhin & Pereira 2010). Lack of resources in these developing countries mean that capacities to handle the effects of climate hazards are low (Laukkonen *et al*. 2009) – this leads to increased vulnerability (Low 2005). Therefore, there is a need to invest in adaptation strategies for the changing climate in developing countries. Mitigation and adaptation strategies are necessary in the management of hazards and disasters which result from climate change (McCarthy 2001). While adaptation strategies help individuals manage, cope and adjust their lives to changing hazards and risks (Smit & Wandel 2006), mitigation strategies aim to reduce the likelihood of disasters occurring, by aiming to change practices which lead to increased production of greenhouses gases (Kane 2000; Prabhakar, Srinivasan & Shaw 2009).

Mitigation and adaptation had always been viewed as separate fields until recently. Both approaches integrated and working together lead to better outcomes for DRR (Nyong *et al*. 2007). The poor are usually the ones most vulnerable to the effects of climate change and a lack of capacity at the community level hinders successful integration of mitigation and adaptation strategies in developing African countries (Michaelowa 2001). Despite these challenges, communities in these developing African countries continue to experience population boom and have survived for generations. Therefore, a necessary entry point to the mitigation or adaptation debate would be to understand how such local communities have used indigenous knowledge to cushion their vulnerability while adapting to the effects of climate change from the past (Nyong *et al*. 2007).

Pastoral farmers in Africa store away fodder as a part of their farming systems. They keep various varieties of herds with varying levels of tolerance to weather conditions. When drought occurs, local farmers rely on cattle for meat as there is less availability of fresh grass, while rearing goats and sheep for other products by feeding them fodder. Through this practice, there is an adaptation to the drought periods is made (Oba 1997). On the other hand, mitigation strategies have helped in reducing emissions and carbon substitution (Nyong *et al*. 2007). Also in ensuring biodiversity, mitigation strategies are usually employed. The World Bank sponsors the development of gene banks for the preservation of local species to be used for breeding in the future. However, there is a need to preserve the indigenous knowledge needed to grow these species in future. Situations such as these highlight the need for development professionals who are more concerned with mitigation strategies, to work with indigenous farmers who usually practice adaptation strategies, to ensure a sustainable future. There is a need to integrate indigenous knowledge in mitigation plans for development, as this knowledge has helped local communities survive and adapt for generations (Nyong *et al*. 2007; Warren 1991).

Focusing on the importance of adaptation strategies towards disaster risk reduction and sustainable development may begin to seem like a narrative; at this stage, it is important to highlight some examples of these local adaptation strategies in action – ironically pointed out by organisations that may employ mitigation strategies in their work on disaster risk reduction and sustainable development. When Cyclone Pam hit the southern hemisphere during 06–15 March 2015, it caused a lot of devastation and loss of lives. *Tearfund*, a non-profit organisation was on ground to help in the wake of the disaster. However, they realised that the number of fatalities related to the cyclone was low. In Vanuatu, even before the onset of the cyclone, a member of the community formed a group comprising of local teenage boys who prepared houses for the cyclone. This group continues to rebuild the houses affected by the cyclone to this day. Another resident of the community hid women and children in an empty baptism pool, with sufficient air and water supply to prevent them being killed by the cyclone. Even after the disaster, a woman in the community gathered unripe bananas which had been uprooted by the cyclone. Instead of allowing the bananas to rot, she used the barks to prepare a local delicacy for her family and provide food for people in the community (Barthow 2015). This shows local adaptation strategies that local communities in that part of the world use in disaster risk reduction and sustainability. Going over the *Red Cross/Red Crescent Societies’* work on the issues of climate change, they highlighted the importance of indigenous early warning strategies that aided the evacuation of almost a million people in India before Cyclone Phailin hit India. However, in Africa, they mentioned various programs – W.M.O. programs, W.H.O. programs, and W.F.P. programs – aimed at disaster risk reduction. They say these programs are undertaken with a combination of expert knowledge and inputs from the local people. Such programs are funded and managed by developed countries (Red Cross/Red Crescent Climate Centre 2013). So why is this so? Why should solutions for disaster risk reduction come from initiatives from developed nations and not from the local people who are directly affected? Where is the local knowledge that had helped the forefathers of Africa survive disasters for centuries?

## Inclusion of indigenous knowledge in disaster risk reduction in Africa: Challenges

Local communities have not always been helpless in the face of hazards and disasters before the advent of scientific knowledge; they have always had their own approaches towards disaster risk reduction using their indigenous knowledge (Gaillard & Mercer 2012). Unfortunately, this knowledge had been widely ignored in preference to scientific knowledge and as Ocholla and Onyancha (2005) noted, this may be as a result of politics as well as ignorance and particular ideologies from scientific knowledge. Scientific researchers are known to work with specific theories and assumptions which have been ‘received’ from previous experts in their various fields – some of these have been applied in the African context because they had attained credibility over the years (Leach & Mearns 1996). However, some of these assumptions have a tendency to alienate other important questions which should be asked, dismissing important evidence of historical data from Africa. An example is the ‘wood fuel crisis’ theory which suggests that cutting of trees for local fuel is the main cause of deforestation in Africa – the fact that most wood used for fuel in Africa are obtained from land clearing for agricultural purposes is brushed aside (Leach & Mearns 1988, 1996). It is important to mention that some of these theories and assumptions were conceived during the period of African colonisation, when the idea of ‘conserving the environment’ was proposed. Such ideas were just used as a form of social control for the benefit of the settlers from Europe. This approach came to be known as colonial science (Fairhead & Leach 1996) and even though the proponents of these ideas had little evidence to support their arguments, these ideas became the guidelines used in agriculture, livestock and forestry management (Beinart 1984; Leach & Mearns 1996; Tiffen, Mortimore & Gichuki 1994). These researchers failed to recognise the relevance of indigenous knowledge systems. Some of the approaches used by these researchers had previously portrayed African locals as agents of environmental change in a negative way, even though not directly (Leach & Mearns 1996).

In the 70s and 80s, famines occurred in the Sahel region as a result of droughts, which ultimately led to the death of about a hundred thousand people due to hunger (Adepoju 2009:3). The West portrayed this disaster as a product of harsh climatic conditions and locusts that ate up plantations. The foreign observers were quick to point out what was termed as ‘inadequate farming practices’ by local farmers in Africa leading to poor harvest. This idea of laying the blame on the local people can be traced back to the colonial period when the locals were accused of ignorance and neglecting their natural resources. To ensure that they manage their environment better, they had to be helped to become civil and modernised (Meier 2007). Colonialism altered the African continent forever. It contributed to a change in the thinking patterns of the locals, as well as affecting the cultural heritage and development of the African people. While some would argue that it brought Western education and Christianity, it is important to note that this form of education was not rooted in ‘African culture and knowledge systems’ – it was education aimed at impacting ‘Western knowledge’. Colonial education, which has graduated to what is now referred to as Western education, destabilised the African indigenous knowledge and technological systems. Locals abandoned their traditional ways of life and indigenous knowledge, which was the core of their ‘indigenousness’, in pursuit of the Western knowledge (Ocheni & Nwankwo 2012). This is one of the reasons why indigenous knowledge is not used on a wider scale in Africa, as the advent of ‘Western knowledge’ in Africa took away indigenous forms of passing down knowledge through generations.

There is also the problem of documentation of indigenous knowledge. African indigenous knowledge tends to be imbedded in people’s memories. It is usually passed down generations through stories told to the younger generation by elders in the local community. However, the newer generation is not interested in learning this knowledge as they are interested in ‘modern’ ways of life. The younger generations are not interested to learn because some believe indigenous knowledge is ‘knowledge of the poor’ (Dei 2000; Kgomotso 2012). Apart from the fact that African indigenous knowledge has not been well documented over time because custodians of this knowledge do not write down this knowledge to be handed to the younger generations, this knowledge also faces the challenge of globalisation as the environment changes every day. There is a breakdown in traditional communication channels and socio-economic imbalances – lifestyle changes and exposure for the younger generation due to global influence limit interaction with the older people in communities and such older people die without passing down the indigenous knowledge (Langill 1999). This leads to greater difficulty as African libraries find it difficult to document this indigenous knowledge to be used for global development initiatives. Lack of funds, technological problems and a lack of specific frameworks from government aimed at harnessing this knowledge also lead to this decline in indigenous knowledge (Sithole 2007). Applying methods which are not well documented in an area such as DRR would pose risks for development and disaster management experts, who would be sceptical to use ideas passed by word-of-mouth from the locals for development policies. This is why DRR policies and initiatives are still carried out using top-down approaches as development experts and even governments believe documented, tested and tried methods are safer to use in Africa. The problem here is that these ideas are usually obtained from different parts of the world and they may not be effectively applied in the African context, leading to failed projects (Swift 1996).

## Conclusion

People are increasingly vulnerable to climate-related hazards the world over. This vulnerability is not evenly distributed as people in less developed countries such as Africa and Asia suffer more human losses. This has led to the argument that disasters are not just a product of weather hazards, but also a product of long-term issues of underdevelopment (Yodmani 2001). Disaster management experts have developed a number of mitigation approaches to help in reducing vulnerability of those most at risk from hazards, but the problem is that these approaches are championed by the top-class within the economy and have led to failures. There is a tendency to ignore the local people who are the ones most directly affected by these hazards. Now, people are beginning to advocate for the implementation of bottom-up approaches which enables local people to use their indigenous knowledge in developing DRR initiatives applicable to their situations (Smit & Wandel 2006; Yodmani 2001). Mitigation strategies used by government and disaster management experts are not doing enough. They fail to recognise how the local community dynamics work. The community-led DRR adaptation initiatives are more suited to the African local people as they create platforms that enable engagement of like minds with shared values and shared knowledge. Locals feel a sense of security and communal belonging when sharing the same knowledge that is indigenous to their culture. This increases the chance of employing participatory approaches to tackle disasters. It has been shown that projects that create effective participation from members of the community yield the best long-term results (Nyong *et al*. 2007; Yodmani 2001).

Local communities should be given the chance to determine their own destinies, to handle their own issues with their own adaptive methods (Sillitoe & Marzano 2008). Local households had always found ways to adapt to their changing environments without the help of DRR experts. However, the focus of government on Western knowledge forms for DRR reduces the confidence of these local households when it comes to expressing adaptation strategies that had worked for them on a larger scale. Government should show more interest in African indigenous knowledge shared and implemented in the most basic form of community (the household unit). There is also a need to understand how these households relate with their environment. It has been shown that the constant interactions between people and their environment always lead to continuous changes in the ways these people relate with the environment (Sillitoe 1998). Each change in climatic conditions leads to different adaptation patterns. Such adaptation has sustained day-to-day life in Africa. Relevant bodies and development initiatives should look to indigenous knowledge as a basic starting point in DRR policies especially at the local level. The knowledge provides the necessary grassroots information, from the historic and cultural contexts, which helps in understanding how things work at the local level – this creates better engagement with local people and better policies. Provisions for documentation of indigenous knowledge should be made in the DRR literature, enabling more researchers to work towards harnessing such knowledge.

## References

[CIT0001] Abam T.K.S, 1993, ‘Bank erosion and protection in Niger Delta’, *Hydrological Sciences* 38(3), 231–241. http://dx.doi.org/10.1080/02626669309492665

[CIT0002] Adepoju A, 2009, ‘Environmental Changes and Migration in the Sahel: An Exploratory Note’, Paper presented at the International Conference: Climate Change and Human Mobility in Africa: Dialogue for a strategic cooperation between Italy and Africa, April 29, 2009, Sala Delle Conferenze Internazionali, Rome, Italy, pp. 1–10.

[CIT0003] Agrawal A, 1995, ‘Dismantling the divide between indigenous and scientific knowledge’, *Development and Change* 26, 413–439. http://dx.doi.org/10.1111/j.1467-7660.1995.tb00560.x

[CIT0004] Arunotai N, 2008, ‘Saved by an old legend and a keen observation: The case of Moken sea nomads in Thailand’, in Shaw R., Uy N., & Baumwoll J. (eds.), *Indigenous knowledge for disaster risk reduction: Good practices and lessons learnt from the Asia-Pacific region*, pp. 73-78, UNISDR Asia and Pacific, Bangkok.

[CIT0005] Barthow J, 2015, ‘Seven amazingly resilient people from Vanuatu I met this week’, viewed 16 June, 2015, from http://www.tearfund.org/en/blog/2015/03/7_amazingly_resilient_people_from_vanuatu/

[CIT0006] Beinart W, 1984, ‘Soil erosion, conservationism and ideas about development: A southern African exploration, 1900–1960’, *Journal of Southern African Studies* 11(1), 52–83. http://dx.doi.org/10.1080/03057078408708088

[CIT0007] Bessant J. & Francis D, 2005, ‘Transferring soft technologies: Exploring adaptive theory’, *International Journal of Technology Management & Sustainable Development* 4(2), 93–112. http://dx.doi.org/10.1386/ijtm.4.2.93/1

[CIT0008] Birkmann J. & von Teichman K, 2010, ‘Integrating disaster risk reduction and climate change adaptation: Key challenges – Scales, knowledge and norm’, *Sustainability Science* 5(2), 171–184. http://dx.doi.org/10.1007/s11625-010-0108-y

[CIT0009] Dei G.S, 2000, ‘Rethinking the role of IKS in the academy’, *International Journal of Inclusive* *Education* 4(2), 111–132. http://dx.doi.org/10.1080/136031100284849

[CIT0010] Dekens J, 2007, ‘Local knowledge for disaster preparedness: A literature review’, *International Centre for Integrated Mountain Development, Kathmandu*, viewed n.d., from http://www.preventionweb.net/publications/view/2693

[CIT0011] Domfeh K.A, 2007, ‘Indigenous knowledge systems and the need for policy and institutional reforms’, *Tribes and Tribals, Indigenous Knowledge Systems and Sustainable Development: Relevance for Africa*, 1(5), 41–52.

[CIT0012] El Sammani M.O. & Dabloub S.M.A, 1996, ‘Making the most of local knowledge: Water harvesting in the Red Sea Hills of northern Sudan’, in Reij C., Scoones I., & Toulmin C. (eds.) *Sustaining the soil: Indigenous soil and water conservation in Africa*, pp. 28–34, Earthscan Publishing Limited, Oxfordshire, United Kingdom.

[CIT0013] Eyong C.T, 2007, ‘Indigenous knowledge and sustainable development in Africa: Case study on Central Africa’, in Boon E.K. & Hens L. (eds.) *Indigenous knowledge systems and development: Relevance for Africa*, pp. 121–139, Kamla-Raj Enterprises, New Delhi, India.

[CIT0014] Eyong C.T., Mufuaya I. & Foy I, 2004, ‘Literature and Culture– The sustainability connection from an African perspective’, in Boon E.K. (ed.) *Regional Sustainable Development Review: Africa*, pp. 1–13, Eolss Publishers, Oxford, United Kingdom.

[CIT0015] Fairhead J. & Leach M, 1996, ‘Rethinking the forest-savanna mosaic: Colonial science & its relics in West Africa’, in Leach M. & Mearns R. (eds.) *The lie of the land: Challenging received wisdom on the African environment*, pp. 105–121, The International African Institute, London.

[CIT0016] Gaillard J.C. & Mercer J, 2012, ‘From knowledge to action: Bridging gaps in disaster risk reduction’, *Progress in Human Geography* 37(1), 93–114. http://dx.doi.org/10.1177/0309132512446717

[CIT0017] Goonatilake S, 1984, *Aborted discovery – Science and creativity in the third world*, Zed Press, London.

[CIT0018] Hilhorst D., Baart J., van der Haar G. & Leeftink F.M, 2015, ‘Is disaster “normal” for indigenous people? Indigenous knowledge and coping practices’, *Disaster Prevention and Management* 24(4), 506–522. http://dx.doi.org/10.1108/DPM-02-2015-0027

[CIT0019] Hiwasaki L., Luna E., Syamsidik & Shaw R, 2014, *Local and indigenous knowledge for community resilience: Hydro-meteorological disaster risk reduction and climate change adaptation in coastal and small inland communities*, UNESCO, Jakarta.

[CIT0020] Jabulani S, 2007, ‘The challenges faced by African libraries information centres in documenting and preserving indigenous knowledge’, *IFLA Journal* 33(2), 117–123. http://dx.doi.org/10.1177/0340035207080304

[CIT0021] Kane S, 2000, ‘Linking adaptation and mitigation in climate change policy’, *Climate Change* 45(1), 75–102. http://dx.doi.org/10.1023/A:1005688900676

[CIT0022] Kelman I., Mercer J. & Gaillard J.C, 2012, ‘Indigenous knowledge and disaster risk reduction’, *Geography* 97(1), 12–21.

[CIT0023] Kgomotso M.H, 2012, ‘Promoting African indigenous knowledge in the knowledge economy’, *Aslib Proceedings* 64(5), 540–554. http://dx.doi.org/10.1108/00012531211263157

[CIT0024] Knapel A., Kitutu M.G., Poesen J., Breugelmans W., Deckers J. & Muwanga A, 2006, ‘Landslides in a densely populated county at the foot slopes of Mount Elgon (Uganda): Characteristics and casual factors’, *Geomorphology* 73(1–2), 149–165. http://dx.doi.org/10.1016/j.geomorph.2005.07.004

[CIT0025] Kulatunga U, 2011, ‘Impact of culture towards disaster risk reduction’, *International Journal of Strategic Property Management* 14(4), 304–313. http://dx.doi.org/10.3846/ijspm.2010.23

[CIT0026] Langill S, 1999, *Indigenous knowledge: A resource kit for sustainable development researchers in dryland Africa*, People, Land and Water Program Initiative, IDRC, Ottawa, Canada, viewed 13 May 2015, from http://www.idrc/plaw/11e-IK.html

[CIT0027] Laukkonen J., Blanco P.K., Lenhart J., Keiner M., Cavric B. & Kinuthia-Njenga C, 2009, ‘Combining climate change adaptation and mitigation measures at the local level’, *Habitat International* 33(3), 287–292. http://dx.doi.org/10.1016/j.habitatint.2008.10.003

[CIT0028] Leach G. & Mearns R, 1988, *Beyond the Woodfuel crisis: People, land and trees*, Earthscan Publications, London.

[CIT0029] Leach M. & Mearns R. (eds.), 1996, *The lie of the land: Challenging received wisdom on the African environment*, The International African Institute, London.

[CIT0030] Low P.S. (ed.), 2005, *Climate change and Africa*, Cambridge University Press, New York.

[CIT0031] McCarthy J.J, 2001, *Climate Change 2001: Impacts, Adaptation and Vulnerability*, Cambridge University Press, New York.

[CIT0032] Meier A, 2007, ‘Natural disasters? Droughts and epidemics in pre-colonial Sudanic Africa’, *The* *Medieval History Journal* 10(1&2), 209–236. http://dx.doi.org/10.1177/097194580701000208

[CIT0033] Melchias G, 2001, *Biodiversity and conservation*, Science Publishers Inc, Enfield.

[CIT0034] Mercer J., Kelman I., Taranis L. & Suchet-Pearson S, 2010, ‘Framework for integrating indigenous and scientific knowledge for disaster risk reduction’, *Disasters* 34(1), 214–239. http://dx.doi.org/10.1111/j.1467-7717.2009.01126.x 19793324 10.1111/j.1467-7717.2009.01126.x

[CIT0035] Michaelowa A, 2001, *Mitigation versus adaptation: The political economy of competition between climate policy strategies and the consequences for developing countries*, HWWA Discussion Paper, 153, Hamburg Institute of International Economics, Hamburg, Germany.

[CIT0036] Mutasa M, 2015, ‘Knowledge apartheid in disaster management discourse: Is marrying indigenous and scientific knowledge the missing link?’, *Jamba: Journal of Disaster Risk Studies* 7(1), Art. #150, 10 pages. 10.4102/jamba.v7i1.150 PMC6014145 29955277

[CIT0037] Mwaura P, 2008, *Indigenous knowledge in disaster management in Africa*, United Nations Environment Programme, Nairobi.

[CIT0038] Nkuinkeu R, 1999, *Medicinal plants and forest exploitation in non-wood forest products of Central Africa: Current research issues and prospects for conservation and development*, FAO Forestry Department, Rome.

[CIT0039] Nyong A., Adesina F. & Osman Elasha B, 2007, ‘The value of indigenous knowledge in climate change mitigation and adaptation strategies in the African Sahel’, *Mitigation and Adaptation Strategies for Global Change* 5, 787–797. http://dx.doi.org/10.1007/s11027-007-9099-0

[CIT0040] Oba G, 1997, *Pastoralists’ traditional drought coping strategies in Northern Kenya: A report for the Government of the Netherlands and the Government of Kenya*, Euroconsult BV, Arnheim and Acacia Consultants Ltd, Nairobi.

[CIT0041] Ocheni S. & Nwankwo B.C, 2012, ‘Analysis of colonialism and impacts in Africa’, *Cross-Cultural Communication* 8(3), 46–54.

[CIT0042] Ocholla D.N. & Onyancha O.B, 2005, ‘The marginalized knowledge: An info-metric analysis of indigenous knowledge publications (1990–2004)’, *SA Libraries and Information Science* 71(3), 247–258.

[CIT0043] Prabhakar S.V.R.K., Srinivasan A. & Shaw R, 2009, ‘Climate change and local level disaster risk reduction planning: Need, opportunity and challenges’, *Mitigation and Adaptation Strategies for Global Change* 4(1), 7–33. http://dx.doi.org/10.1007/s11027-008-9147-4

[CIT0044] Rahman A. & Talukder F.A, 2006, ‘Bioefficacy of some plant derivatives that protect grain against the pulse beetle, *Callosobruchus maculatus*’, *Journal of Insect Science* 6(3), 1–10. http://dx.doi.org/10.1673/1536-2442(2006)6[1:BOSPDT]2.0.CO;2 10.1673/1536-2442(2006)6[1:BOSPDT]2.0.CO;2 PMC2990289 19537990

[CIT0045] Red Cross/Red Crescent Climate Centre, 2013, *Annual Report 2013: Innovation, participation and learning in climate risk management*, viewed 30 May 2015, from http://www.climatechange.org/downloads/files/RCCC_AR-2013

[CIT0046] Rist S. & Dahdouh-Guebas F, 2006, ‘Ethnosciences – A step towards the integration of scientific and indigenous forms of knowledge in the management of natural resources for the future’, *Environment, Development and Sustainability* 8(4), 467–493.

[CIT0047] Rybcynzski W, 1991, *Paper heroes, appropriate technology: Panacea or pipedream*, Penguin, New York.

[CIT0048] Shackleton S. & Campbell B.M, 2000, *Re-empowering communities to manage natural resources: Where does the new power lie? Case studies from Southern and Eastern Africa*, SADC Natural Resource Management Project (CIFOR/WWF), Harare.

[CIT0049] Shaw R., Noralene U. & Baumwoll J, 2008a, *Indigenous knowledge for disaster risk reduction: Good practices and lessons learned from experiences in the Asia-Pacific region*, United Nations International Strategy for Disaster Risk Reduction (UNISDR), Bangkok.

[CIT0050] Shaw R., Pulhin J.M. & Pereira J.J. (eds.), 2010, *Climate change adaptation and disaster risk reduction: An Asian perspective*, Emerald, Bingley, England.

[CIT0051] Shaw R., Takeuchi Y., Uy N. & Sharma A, 2008b, *Indigenous knowledge: Disaster risk reduction, policy note*, United Nations International Strategy for Disaster Risk Reduction, Kyoto, Japan.

[CIT0052] Sillitoe P, 1998, ‘Knowing the land: Soil and land resource evaluation and indigenous knowledge’, *Soil Use and Management* 14, 188–193.

[CIT0053] Sillitoe P. & Marzano M, 2008, ‘Future of indigenous knowledge research in development’, *Futures* 41, 13–23. http://dx.doi.org/10.1016/j.futures.2008.07.004

[CIT0054] Sithole J, 2007, ‘The challenges faced by African libraries and information centres in documenting and preserving indigenous knowledge’, *IFLA Journal* 33(2), 117–123. http://dx.doi.org/10.1177/0340035207080304

[CIT0055] Smit B. & Wandel J, 2006, ‘Adaptation, adaptive capacity and vulnerability’, *Global Environmental Change* 16, 282–292. http://dx.doi.org/10.1016/j.gloenvcha.2006.03.008

[CIT0056] Swift J, 1996, ‘Desertification: Narratives, winners and losers’, in Leach M. & Mearns R. (eds.) *The lie of the land: Challenging received wisdom on the African environment*, pp. 73–90, The International African Institute, London.

[CIT0057] Tharakan J, 2015, ‘Indigenous knowledge systems – A rich appropriate technology resource’, *African Journal of Science, Technology, Innovation and Development* 7(1), 52–57. http://dx.doi.org/10.1080/20421338.2014.987987

[CIT0058] Tiffen M., Mortimore M. & Gichuki F, 1994, *More people, less erosion: Environmental recovery in Kenya*, Wiley, Chichester.

[CIT0059] UNISDR, 2005, *Building the resilience of nations and communities to disaster: An introduction to the Hyogo Framework for Action*, UNISDR, Geneva.

[CIT0060] Warren D.M, 1991, *Using indigenous knowledge in agricultural development*, World Bank Discussion Paper, No. 127, The World Bank, Washington.

[CIT0061] Yodmani S, 2001, ‘Disaster management and vulnerability reduction: Protecting the poor’, Paper presented at the Social Protection Workshop 6: Protecting Communities – Social Funds and Disaster Management, under the Asia and Pacific Forum on Poverty Reduction, held at the Asian Development Bank, Manila, Philippines, 05–09 February, 2011, pp. 1–32.

[CIT0062] Zhou B., Fu M., Xie J., Yang X. & Li Z, (2005), ‘Ecological functions of bamboo forest: Research and application’, *Journal of Forestry Research* 16(2), 143–147. http://dx.doi.org/10.1007/BF02857909

[CIT0063] Zhouying J, 2005, *Global technological change: From hard technology to soft technology*, Intellect Books, Chicago, USA.

